# Effect of incision on visual outcomes after implantation of a trifocal diffractive IOL

**DOI:** 10.1186/s12886-018-0846-7

**Published:** 2018-07-13

**Authors:** Shasha Xue, Guiqiu Zhao, Xiaoni Yin, Jing Lin, Cui Li, Liting Hu, Lin Leng, Xuejiao Yang

**Affiliations:** The Affiliated Hospital of Qingdao University, No.16, Jiangsu Road, Qingdao, China

**Keywords:** Cataract, Incision, Trifocal IOL, Corneal astigmatism

## Abstract

**Background:**

To evaluate visual acuity, corneal astigmatism and corneal higher-order aberrations (HOAs) after implantation of trifocal diffractive IOLs operated with either a corneal steep-axis incision or 135° incision.

**Method:**

This prospective study enrolled patients randomly assigned to different groups. According to preoperative corneal astigmatism, 101 eyes of 77 patients were assigned into group A_1_ (0 ~ 0.50 D) or A_2_ (0.51 ~ 1.00 D) with a corneal steep-axis incision or group B_1_ (0 ~ 0.50 D) or B_2_ (0.51 ~ 1.00 D) with a 135° incision. Visual acuity, corneal astigmatism and corneal higher-order aberrations (HOAs) were followed-up for 3 months.

**Results:**

Corneal astigmatism in group A_2_ significantly decreased 3 months after surgery (*P* < 0.01) and was significantly lower than that in group B_2_ 1 day, 2 weeks, 1 month, and 3 months postoperatively (all values of P < 0.01). The following parameters were better in group A_2_ than in group B_2_: uncorrected intermediate visual acuity (UIVA) at 1 day, 2 weeks, 1 month, and 3 months (*P* = 0.00, 0.00, 0.01, 0.01, respectively);uncorrected distance visual acuity (UDVA) at 1 day and 2 weeks (*P* = 0.00, 0.01); and uncorrected near visual acuity (UNVA) at 1 day, 2 weeks, and 1 month postoperatively (*P* = 0.00, 0.01, 0.02, respectively).

**Conclusions:**

After a corneal steep-axis incision, patients with preoperative corneal astigmatism of 0.51 D to 1.00 D exhibited reduced corneal astigmatism and achieved better UIVA and early postoperative UDVA/UNVA.

**Trial registration:**

Retrospectively Registered Trials ISRCTN10086721, 23/06/2018.

## Background

Patients’ expectations regarding refractive outcomes and spectacle independence have increased substantially, and both cataract patients and refractive patients have the same demands [1]. Multifocal IOLs were developed with the target of reducing spectacle dependence, which can provide patients with near and distance visual restoration after cataract surgery [2]. However, intermediate vision is limited because no specific focus is provided for this distance. Trifocal diffractive IOL designs have shown their capability to provide effective uncorrected intermediate visual acuity (UIVA) restoration without degradation of uncorrected distance visual acuity (UDVA) or uncorrected near visual acuity (UNVA). This new concept of IOL has confirmed good performance for visual outcomes, patient satisfaction and spectacle independence [3–10].

Patients’ preoperative corneal astigmatism is critical to the choice of trifocal diffractive IOL, which is a key factor influencing the visual acuity and refractive outcomes postoperatively. Many studies have shown that the location of the corneal incision has an impact on postoperative corneal astigmatism and higher-order aberrations (HOAs), such as degradation of vision at night, halos and glare [11]. However, there is no research on the effect of incisions on visual outcomes after implantation of trifocal diffractive IOLs. This study aimed to evaluate visual acuity, corneal astigmatism and corneal HOAs after implantation of a trifocal diffractive IOL operated with either a corneal steep-axis incision or a 135° incision.

## Methods

### Patients

In this prospective comparative study, 101 eyes of 77 patients undergoing cataract surgery with implantation of a trifocal diffractive IOL (AT LISA tri 839MP, Carl Zeiss Meditec, Germany) at the Affiliated Hospital of Qingdao University between January 2016 and December 2017 were enrolled. All eyes were divided into two groups: group A including 49 eyes of 37 patients with a 2.8 mm clear corneal incision at the steep-axis and group B including 52 eyes of 40 patients with a 2.8 mm clear corneal incision at 135°. According to the preoperative corneal astigmatism, groups A and B were separated into two subgroups: A_1_ (0 ~ 0.50 D with 22 eyes), A_2_ (0.51 ~ 1.00 D with 27 eyes), B_1_ (0 ~ 0.50 D with 23 eyes), and B_2_ (0.51 ~ 1.00 D with 29 eyes).The inclusion criteria were cataract or presbyopia patients who had preexisting corneal astigmatism of less than 1.00 D and seeking spectacle independence suitable for refractive lens exchange. The exclusion criteria were patients with a history of previous ocular surgery or ocular diseases, such as ocular inflammation, keratopathy, glaucoma, retinopathy or optic neuropathy.

The research adhered to the tenets of the Declaration of Helsinki and was approved by the ethics committee of the Affiliated Hospital of Qingdao University. A consent form were signed by all patients who were adequately informed and voluntary participated in the study.

### Examination protocol

Complete preoperative and 1-day, 2-week, 1-month, and 3-month postoperative ophthalmological examinations were performed in all cases, including monocular visual acuity (logMAR), Goldmann applanation tonometry, slit-lamp examination, funduscopy, manifest refraction, optical biometry (IOL Master 500; Carl Zeiss Meditec), and measurement of total corneal astigmatism and corneal aberration (both with a Galilei G2, Ziemer ophthalmic systems AG, Port, Switzerland). Visual acuities including preoperative corrected distance visual acuity (CDVA), postoperative UDVA, UIVA and UNVA were measured. The classifications of astigmatic axial length with the rule (WTR) (90° ± 30°), against the rule (ATR) (0°to 30°or 150°to 180°), and oblique (30°to 60°or 120°to 150°) were used. The calculation of surgically induced astigmatism (SIA) adopted the Jaffe/Clayman vector analysis [12]. The corneal aberrations considered a pupil aperture of 3.5 mm and were calculated and recorded with the Zernike coefficient.

### Surgical procedure

All surgeries were performed by the same experienced surgeon who was masked to the patients’ data before the surgery. Sutureless 2.8-mm main corneal incisions either on the corneal steep-axis or at 135° were set up in the navigation system by the same experimenter prior to the surgical procedure. After manual capsulorhexis and phacoemulsification, the trifocal diffractive IOL was inserted into the capsular bag through the main corneal incision using a specific injector. A postoperative topical therapy based on a combination of levofloxacin, nebcin and dexamethasone eye drops were prescribed to be applied four times daily for 1 week.

### Intraocular lens

The AT Lisa tri 839MP is a diffractive trifocal preloaded IOL with a 6.0 mm biconvex optic, an overall length of 11.0 mm, and a posterior surface with a sphericity of − 0.18 μm. The near add is + 3.33D, and the intermediate add is + 1.66D. Its design allocates 50% of light to far, 20% to intermediate, and 30% to near vision. The central 4.34 mm follows the described trifocal design, and the peripheral part is only bifocal.

### Statistical analysis

SPSS statistics software package version 22.0 was used for statistical analysis. Kolmogorov–Smirnov test was used to check the normality of the data distribution. When parametric analysis was possible, Student’s t-tests for paired data were performed for all parameter comparisons. Otherwise, the Wilcoxon signed rank test was applied to assess the significance of differences between examinations. A power analysis was performed with G*Power software, and figures were made by GraphPad Prism. In all cases, the same level of significance (*P* < 0.05) was used.

## Results

The study enrolled 101 eyes of 77 patients with a mean age of 59.33 years ranging from 43.00 to 77.00 years. There was no significant difference in age between the groups. The mean preoperative anterior chamber depth (ACD) and axial length (AL) were 3.21 mm (standard deviation [SD]: 0.40; median: 3.33; range: 2.54 to 3.94 mm) and 23.99 mm (SD: 1.42; median: 23.54; range: 21.91 to 27.51 mm), respectively. There were no statistically significant differences in preoperative ACD or AL between groups (Table 1).

**Table 1:** Preoperative and Postoperative Clinical Date

Parameters *mean ± SD* *median (range)*	Group A_1_	Group B_1_	A_1_/B_1_ (*P*)	Group A_2_	Group B_2_	A_2_/B_2_ (*P*)
Age (y)	59.23 ± 7.12 57.00 (51.00 to 74.00)	61.52 ± 7.86 62.00 (43.00 to 77.00)	0.31	57.22 ± 10.86 55.00(51.00 to 76.00)	59.62 ± 7.54 59.00 (43.00 to 75.00)	0.28
ACD (mm)	3.15 ± 0.36 3.26 (2.60 to 3.78)	3.24 ± 0.41 3.34 (2.54 to 3.81)	0.45	3.29 ± 0.41 3.36 (2.54 to 3.94)	3.17 ± 0.43 3.33 (2.54 to 3.78)	0.89
AL (mm)	23.68 ± 1.16 23.49 (21.96 to 26.45)	24.29 ± 1.53 24.37 (22.00 to 26.84)	0.14	24.21 ± 1.56 24.44 (21.91 to 27.51)	23.78 ± 1.36 23.53 (21.96 to 26.74)	0.30
Preoperative UDVA	0.52 ± 0.18 0.52 (0.22 to 1.00)	0.58 ± 0.16 0.60 (0.30 to 0.82)	0.23	0.56 ± 0.16 0.60 (0.22 to 0.82)	0.56 ± 0.17 0.52 (0.22 to 0.92)	0.85
Preoperative CDVA	0.40 ± 0.14 0.40 (0.10 to 0.70)	0.47 ± 0.12 0.40 (0.20 to 0.70)	0.24	0.49 ± 0.15 0.52 (0.22 to 0.82)	0.50 ± 0.18 0.52 (0.10 to 0.82)	0.34
Spherical Refraction^*a*^ (D)	−0.11 ± 0.21 0.00 (− 0.50 to 0.25)	−0.13 ± 0.24 0.00 (− 0.50 to 0.25)	0.67	− 0.07 ± 0.25 0.00 (− 0.50 to 0.25)	−0.07 ± 0.23 0.00 (− 0.50 to 0.25)	0.94
Cylindrical Refraction^*a*^ (D)	−0.34 ± 0.21 − 0.38 (− 0.75 to 0.00)	−0.37 ± 0.24 − 0.50 (− 0.75 to 0.00)	0.81	−0.34 ± 0.16 − 0.25 (− 0.50 to 0.00)	−0.54 ± 0.25 − 0.50 (− 1.00 to 0.00)	0.00
SE^*a*^ (D)	−0.28 ± 0.22 − 0.25 (− 0.63 to 0.25)	−0.32 ± 0.23 − 0.38 (− 0.75 to 0.13)	0.59	−0.25 ± 0.24 − 0.25 (− 0.63 to 0.25)	−0.34 ± 0.26 − 0.38 (− 1.00 to 0.13)	0.18

^a^3 months postoperation

### Corneal astigmatism

There were no statistically significant differences between 3-month postoperative and preoperative corneal astigmatism in groups A_1_, B_1_, or B_2_ (*P* = 0.17, 0.15, 0.22, respectively). However, corneal astigmatism in group A_2_ 3 months postoperatively was significantly lower than preoperatively (*P* < 0.01). There were no statistically significant differences between group A_1_ and group B_1_ 1 day, 2 weeks, 1 month, or 3 months postoperatively (*P* = 0.32, 0.73, 0.42, 0.29, respectively), but corneal astigmatism in group A_2_ was significantly lower than group B_2_1 day, 2 weeks, 1 month, and 3 months postoperatively (all *P* < 0.01) (Table 2).

**Table 2:** Preoperative and Postoperative Corneal Astigmatism Data (D)

Groups *mean ± SD median (range)*	Preoperation	1 Day Postoperation	2 Weeks Postoperation	1 Month Postoperation	3 Months Postoperation	*P* ^*a*^
A_1_	0.37 ± 0.07 0.39 (0.23 to 0.49)	0.68 ± 0.32 0.82 (0.20 to 1.26)	0.64 ± 0.22 0.60 (0.26 to 1.05)	0.55 ± 0.17 0.55 (0.25 to 0.86)	0.42 ± 0.12 0.44 (0.22 to 0.61)	0.17 (power = 50.64%)
A_2_	0.73 ± 0.11 0.70 (0.58 to 0.99)	0.63 ± 0.26 0.66 (0.26 to 1.27)	0.48 ± 0.12 0.45 (0.29 to 0.86)	0.49 ± 0.09 0.54 (0.36 to 0.60)	0.44 ± 0.09 0.45 (0.25 to 0.56)	0.00 (power = 100%)
B_1_	0.40 ± 0.07 0.41 (0.27 to 0.49)	0.80 ± 0.46 0.88 (0.20 to 1.87)	0.66 ± 0.23 0.62 (0.26 to 1.00)	0.60 ± 0.22 0.57 (0.25 to 1.00)	0.47 ± 0.18 0.53 (0.12 to 0.88)	0.15 (power = 52.65%)
B_2_	0.73 ± 0.12 0.68 (0.58 to 0.99)	1.01 ± 0.38 0.91 (0.66 to 2.16)	0.79 ± 0.30 0.69 (0.48 to 1.54)	0.79 ± 0.28 0.83 (0.45 to 1.83)	0.69 ± 0.21 0.70 (0.43 to 1.35)	0.22 (power = 31.10%)
A_1_/B_1_ (*P*)	0.21 (power = 40.89%)	0.32 (power = 25.94%)	0.73 (power = 8.83%)	0.42 (power = 21.03%)	0.29 (power = 28.57%)	–
A_2_/B_2_ (*P*)	0.98 (power = 5.00%)	0.00 (power = 99.61%)	0.00 (power = 99.96%)	0.00 (power = 99.99%)	0.00 (power = 100%)	–

^a^Comparison between preoperation and 3 months postoperation

**Fig. 1:**
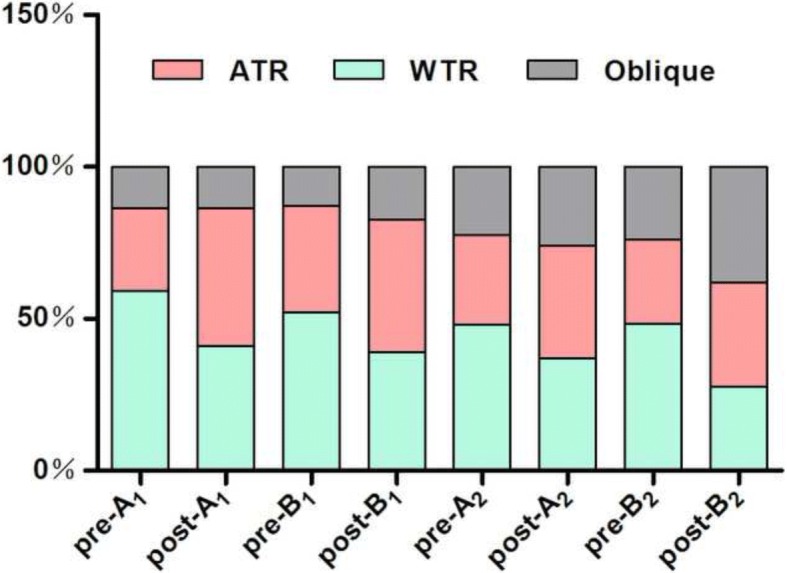
Preoperative and postoperative proportion of WTR, ATR, Oblique in each subgroup

The proportion of WTR in group A_1_declined from 59.1% preoperatively to40.9% 3 months postoperatively, while ATR increased from 27.3 to 45.5%.The WTR of group B_1_ decreased from 52.2 to 39.1%, while ATR increased from 34.8 to 43.5%. The WTR of group A_2_ decreased from 48.1 to 37.0%, while ATR increased from 29.6 to 37.1%.The WTR of group B_2_ decreased from 48.3 to 27.6%, while ATR increased from 27.6 to 34.5%. However, the oblique of group B_2_ increased from 24.1% preoperatively to 37.9% 3 months postoperatively, but no obvious changes were found in groups A_1_, B_1_, or A_2_ (13.6 to 13.6%, 13.0 to 17.4%, 22.2 to 25.9%, respectively). (Fig. 1).

**Fig. 2:**
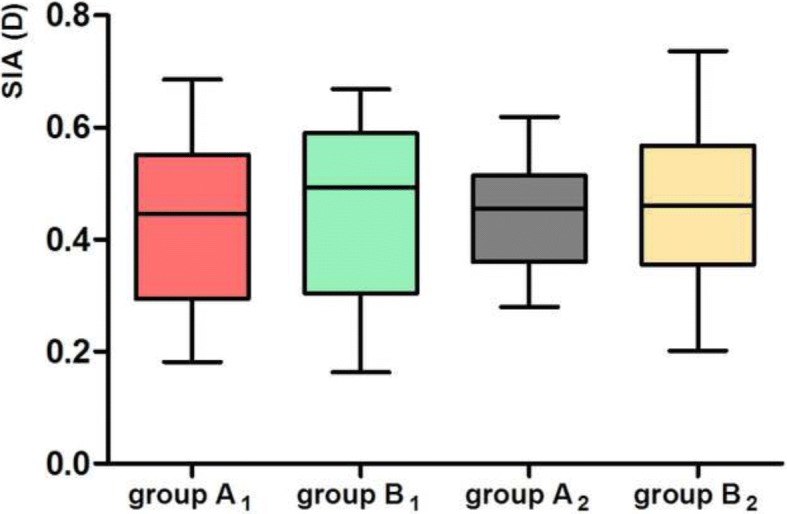
SIA at 3 months postoperation in group A_1_, B_1_, A_2_, B_2_

No significant differences in surgically induced astigmatism (SIA) were detected between group A_1_ and group B_1_ nor between group A_2_ and group B_2_3 months postoperatively (*P* = 0.61, 0.82, respectively). (Fig. 2).

### Visual acuity

There were no significant differences in preoperative UDVA and CDVA between the subgroups (Table 1). Postoperative visual acuity in each group was definitely better than preoperatively. No statistically significant differences in UDVA, UIVA, or UNVA between group A_1_ and B_1_ were found 1 day, 2 weeks, 1 month, or 3 months postoperatively (all values of *P* > 0.05). However, the UIVA of group A_2_ was significantly better than that of group B_2_1 day, 2 weeks, 1 month, and 3 months postoperatively (*P* = 0.00, 0.00, 0.01, 0.01, respectively), while UDVA 1 day and 2 weeks (*P* = 0.00, 0.01) and UNVA 1 day, 2 weeks, and 1 month postoperatively (*P* = 0.00, 0.01, 0.02, respectively)in group A_2_ were better than those in group B_2_.However, there were no significant differences in UDVA 1 month or 3 months postoperatively (*P* = 0.26, 0.44) or in UNVA 3 months postoperatively (*P* = 0.45) (Table 3).

**Table 3:** Postoperative Visual Acuity Data (logMAR)

Visions *mean ± SD median (range)*	Group A_1_	Group B_1_	A_1_/B_1_ (*P*)	Group A_2_	Group B_2_	A_2_/B_2_ (*P*)
UDVA (1 day)	0.06 ± 0.05 0.10 (0.00 to 0.15)	0.03 ± 0.06 0.00 (− 0.08 to 0.20)	0.11 (power = 55.88%)	−0.01 ± 0.04 0.00 (− 0.08 to 0.10)	0.06 ± 0.08 0.00 (0.00 to 0.22)	0.00 (power = 99.27%)
UDVA (2 weeks)	0.01 ± 0.06 0.00 (− 0.08 to 0.10)	0.00 ± 0.05 0.00 (− 0.08 to 0.10)	0.72 (power = 14.75%)	−0.01 ± 0.03 0.00 (− 0.08 to 0.05)	0.02 ± 0.05 0.00 (− 0.08 to 0.10)	0.01 (power = 85.12%)
UDVA (1 month)	−0.01 ± 0.04 0.00 (− 0.08 to 0.05)	0.00 ± 0.05 0.00 (− 0.08 to 0.10)	0.65 (power = 17.99%)	−0.01 ± 0.03 0.00 (− 0.08 to 0.05)	0.00 ± 0.05 0.00 (− 0.08 to 0.10)	0.26 (power = 22.68%)
UDVA (3 months)	−0.02 ± 0.04 0.00 (− 0.08 to 0.05)	−0.01 ± 0.04 0.00 (− 0.08 to 0.05)	0.40 (power = 20.62%)	−0.01 ± 0.03 0.00 (− 0.08 to 0.00)	0.00 ± 0.05 0.00 (− 0.08 to 0.10)	0.44 (power = 22.68%)
UIVA (1 day)	0.10 ± 0.06 0.10 (0.00 to 0.22)	0.09 ± 0.06 0.10 (0.00 to 0.20)	0.59 (power = 13.68%)	0.03 ± 0.05 0.00 (0.00 to 0.15)	0.12 ± 0.11 0.10 (0.00 to 0.30)	0.00 (power = 98.76%)
UIVA (2 weeks)	0.06 ± 0.05 0.10 (− 0.08 to 0.10)	0.03 ± 0.06 0.00 (− 0.08 to 0.20)	0.14 (power = 55.88%)	0.02 ± 0.04 0.00 (0.00 to 0.10)	0.07 ± 0.05 0.10 (0.00 to 0.15)	0.00 (power = 99.25%)
UIVA (1 month)	0.03 ± 0.05 0.00 (− 0.08 to 0.15)	0.02 ± 0.05 0.00 (− 0.08 to 0.15)	0.55 (power = 16.24%)	0.02 ± 0.03 0.00 (0.00 to 0.10)	0.07 ± 0.08 0.10 (− 0.08 to 0.20)	0.01 (power = 92.08%)
UIVA (3 months)	0.01 ± 0.03 0.00 (− 0.08 to 0.05)	0.01 ± 0.04 0.00 (− 0.08 to 0.10)	0.57 (power = 5.00%)	0.00 ± 0.04 0.00 (− 0.08 to 0.10)	0.04 ± 0.06 0.05 (− 0.08 to 0.10)	0.01 (power = 89.46%)
UNVA (1 day)	0.11 ± 0.07 0.10 (0.00 to 0.20)	0.12 ± 0.05 0.10 (0.00 to 0.20)	0.50 (power = 13.52%)	0.10 ± 0.05 0.10 (0.00 to 0.20)	0.19 ± 0.09 0.20 (0.10 to 0.30)	0.00 (power = 99.82%)
UNVA (2 weeks)	0.08 ± 0.04 0.10 (0.00 to 0.15)	0.09 ± 0.03 0.10 (0.00 to 0.15)	0.22 (power = 23.84%)	0.10 ± 0.05 0.10 (0.00 to 0.20)	0.14 ± 0.05 0.10 (0.00 to 0.20)	0.01 (power = 90.47%)
UNVA (1 month)	0.06 ± 0.06 0.05 (0.00 to 0.15)	0.08 ± 0.03 0.10 (0.00 to 0.10)	0.19 (power = 40.00%)	0.09 ± 0.04 0.10 (0.00 to 0.15)	0.12 ± 0.05 0.10 (0.00 to 0.20)	0.02 (power = 78.86%)
UNVA (3 months)	0.05 ± 0.06 0.05 (− 0.08 to 0.15)	0.04 ± 0.05 0.00 (− 0.08 to 0.10)	0.59 (power = 14.75%)	0.09 ± 0.05 0.10 (0.00 to 0.20)	0.10 ± 0.05 0.10 (0.00 to 0.20)	0.45 (power = 18.24%)

### Corneal aberration

**Fig. 3:**
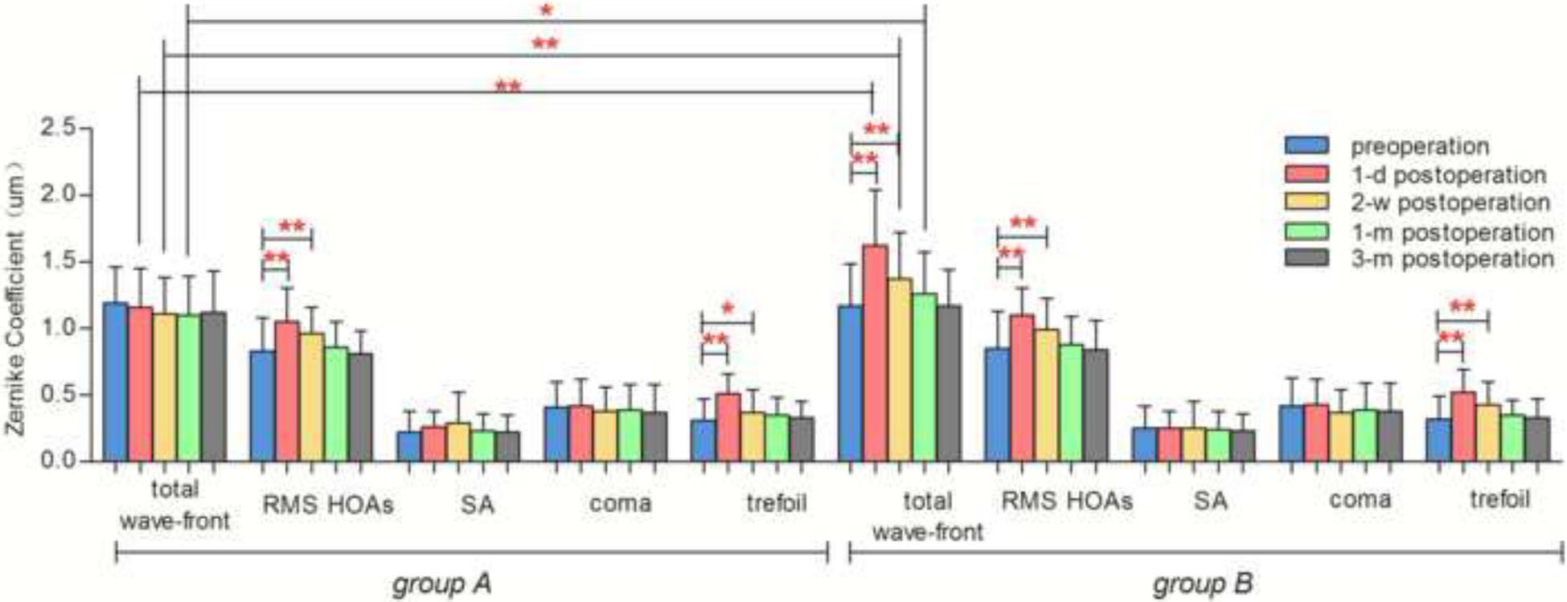
Preoperative and postoperative total corneal wave-front, RMS HOAs, SA, coma, trefoil in each subgroup, * *P < 0.05*, ** *P < 0.01*

There were no significant differences in preoperative total corneal wave-front aberration, root mean square value of corneal higher-order aberrations (RMS HOAs), spherical aberration (SA), coma, or trefoil between group A and group B. Total corneal wave-front aberrations were much higher 1 day, 2 weeks, and 1 month postoperatively in group B than in group A (all *P* < 0.01). There were no statistically significant differences in total corneal wave-front aberrations 3 months postoperatively or in RMA HOAs, SA, coma, or trefoil 1 day, 2 weeks, 1 month, and 3 months postoperatively between group A and group B (all *P* > 0.05). (Fig. 3).

In group A, RMS HOAs and trefoil 1 day and 2 weeks postoperatively increased apparently (all *P* < 0.05), while there were no differences in RMS HOAs or trefoil 1 month or 3 months postoperatively. There were no obvious changes in total corneal wave-front aberrations, SA or coma after surgery (all *P* > 0.05). (Fig. 3).

In group B, the level of total corneal wave-front aberrations, RMS HOAs, and trefoil 1 day and 2 weeks postoperatively (all values of P < 0.01) significantly increased, but there were no differences 1 month or 3 months postoperatively. There were no obvious changes in SA or coma after surgery (all *P* > 0.05). (Fig. 3).

## Discussion

The trifocal diffractive IOLs have shown perfect visual restoration of intermediate vision without degradation of distance or near vision. It’s worth noting that its efficacy is affected by many factors, among which incision location, SIA and preoperative corneal astigmatism are of great importance. Owing to personal surgical practice, some surgeons tend to choose a habitual incision location. Mojzis et al. [13] adopted temporal clear corneal incision, Florian et al. [14] chose incision at the corneal steep-axis, and Matthias Müller et al. [1] used incisions at twelve o’clock on the cornea. Effective restoration of postoperative distance vision, intermediate vision, and near vision was obtained in their studies. However, the influence of the location of corneal incision on postoperative residual corneal astigmatism and visual acuities after implantation of the trifocal diffractive IOLs was unclear. Therefore, we comprehensively assessed the performance of a steep-axis incision and 135° incision. We found that after a corneal steep-axis incision, patients with preoperative corneal astigmatism of 0.51 D to 1.00 D achieved reduced corneal astigmatism and better UIVA and early postoperative UDVA/UNVA.

In our study, we found that when patients’ preoperative corneal astigmatism was under 0.50 D, there were no significant differences in postoperative vision or corneal astigmatism between a steep-axis incision and 135° incision. Because currently used incision sizes are small, even on the micro scale, their interference with the cornea is not significant. Therefore, surgeons can select an appropriate incision based upon their own operational preferences. As for patients with preoperative corneal astigmatism of 0.51 D to 1.00 D, making a corneal incision at the steep-axis can reduce postoperative corneal astigmatism and provide patients with better UIVA and better early postoperative UDVA/UNVA. This kind of trifocal IOL design allocates 50% of light to far, 20% to intermediate and 30% to near vision. Owing to the proportion of light allocation, the influence of corneal astigmatism on visual acuity after implantation with the trifocal diffractive IOLs is UDVA > UNVA > UIVA, but the sensitivity of corneal astigmatism is UIVA > UNVA > UDVA.

Corneal astigmatism was much higher in the early period after the surgery due to corneal edema, but the steep-axis incision shaped the cornea more regularly than the 135° incision after cataract operation. This difference produced early postoperative UDVA, UIVA, and UNVA that were much better in patients with corneal steep-axis incision. With the healing of the corneal incision and fading of corneal edema, corneal astigmatism reduced and gradually became steady, and intermediate vision was more susceptible to corneal astigmatism, which maybe small but is indeed important for UIVA. This study is the first clinical study comparing the visual outcomes obtained with either corneal steep-axis incision or 135° incision and showing the realistic benefits of corneal steep-axis incision in patients implanted with trifocal diffractive IOLs.

There were no significant differences in the SIA between a steep-axis incision and 135° incision in our study. Postoperative astigmatism depends on preoperative corneal astigmatism and SIA. Since incisions currently are small or even on a micro scale in cataract surgery, the value of SIA is not large enough to markedly affect visual acuity [11]. There is an inseparable relationship between corneal astigmatism and vision quality after implantation with a trifocal diffractive IOL. The recommended corneal astigmatism from cataract surgery with trifocal diffractive IOL is no more than 0.75 D. However, Elizabeth et al. [5] chose patients with preoperative corneal astigmatism under 1.00 D, and Peter Mojzis [15] and Florian Tobias selected 1.25 D [16] at most, but Thomas et al. [17] expanded the range to 1.50 D. Our study opted for preoperative corneal astigmatism equal to or less than 1.00 D. Excellent visual outcomes were obtained, and a significant improvement in UDVA, UIVA, and UNVA was found in all these studies with appropriate incision locations. Whether there is an acceptable range of preoperative corneal astigmatism with implantation of trifocal diffractive IOLs needs further study.

We found a drift phenomenon from WTR to ATR postoperatively with a corneal steep-axis incision and135° incision. This finding was consistent with Cui Y’s [18, 19] studies, which showed the same phenomenon after cataract surgery. However, the impact of the astigmatic axial on visual acuity was oblique> ATR > WTR astigmatism [20]. In our study, a higher percentage of oblique astigmatism but a much lower percentage of WTR astigmatism was found postoperatively in group B_2_ compared with those in group A_2_. This finding maybe another factor that contributed to better outcomes in group A_2_ than in group B_2_.

We detected that there was no difference in corneal HOAs between steep-axis incision and 135° incision, but both corneal HOAs from the different incisions were much higher in the early period after the surgery. Some patients achieved vision of 0.00 logMAR or better but still suffered from degradation vision at night, halos and glare. This phenomenon may be due to the increase in postoperative HOAs. Mojzis et al. [3] reported that after surgery for trifocal diffractive IOLs, there was a significant decrease in ocular aberrations and internal aberrations, while there was no statistically significant difference between preoperative and postoperative corneal aberrations. In our study, corneal HOAs increased with steep-axis and 135° incisions due to the existence of a surgical incision and early postoperative corneal edema. However, it reduced gradually and was not different compared to preoperative HOAs 3 months postoperatively. This finding was consistent with Florian T.A Kretz’s discovery [14] that negative effects were not disturbing and were a temporary phenomenon that reduced over time. However, total corneal wave-front aberration was much higher with the 135° incision because of its larger, early postoperative corneal astigmatism than that with the steep-axis incision that shaped the cornea more regularly.

## Conclusions

In summary, steep-axis incision may be an ideal incision choice for patients with preoperative corneal astigmatism of 0.51 D to 1.00 D for trifocal diffractive IOL implantation. However, for patients with preoperative corneal astigmatism under 0.50 D, surgeons can select the appropriate incision based on their own preferences.

## Abbreviations

ACD: Anterior chamber depth
AL: Axial length
ATR: Against the rule
CDVA: Corrected distance visual acuity
HOAs: Higher-order aberrations
RMS HOAs: Root mean square value of corneal higher-order aberrations
SA: Spherical aberration
SE: Spherical equivalent
SIA: Surgically induced astigmatism
UDVA: Uncorrected distance visual acuity
UIVA: Uncorrected intermediate visual acuity
UNVA: Uncorrected near visual acuity
WTR: With the rule
